# ﻿*Triplophysadaryoae*, a new nemacheilid loach species (Teleostei, Nemacheilidae) from the Syr Darya River basin, Central Asia

**DOI:** 10.3897/zookeys.1125.85431

**Published:** 2022-10-19

**Authors:** Bakhtiyor Sheraliev, Yorkinoy Kayumova, Zuogang Peng

**Affiliations:** 1 Key Laboratory of Freshwater Fish Reproduction and Development (Ministry of Education), Southwest University, School of Life Sciences, Chongqing 400715, China Southwest University Chongqing China; 2 Fergana State University, Faculty of Life Sciences, Fergana 150100, Uzbekistan Fergana State University Fergana Uzbekistan

**Keywords:** Fergana Valley, freshwater fish, ichthyofauna, phylogeny, taxonomy

## Abstract

*Triplophysadaryoae*, new species, is described from the Sokh River, a former tributary of Syr Darya that today fails to reach the river, in the Sokh District, an exclave of Uzbekistan, surrounded by Kyrgyzstan. *Triplophysadaryoae* is distinguished from other species of *Triplophysa* in Central Asia by a truncate caudal fin with 13 or 14 branched rays, body without obvious mottling, dorsal-fin origin opposite to pelvic-fin insertion, and absence of the posterior chamber of the air bladder. Molecular data suggest that *Triplophysadaryoae* is closely related to *T.ferganaensis* from the Shakhimardan stream, a small tributary of Syr Darya in the Yordon village, another exclave of Uzbekistan in Kyrgyzstan. The two species were separated by a Kimura 2-parameter genetic distance of 2.8% in the mitochondrial DNA cytochrome *c* oxidase subunit I barcode region; they are also distinguished morphologically. A key to the species of *Triplophysa* in the Syr Darya basin and adjacent regions is provided.

## ﻿Introduction

The genus *Triplophysa* Rendahl, 1933, comprises approximately 160 species (Fricke et al. 2022). The genus is widespread in western and central Asian waters, inland drainages of Balochistan, northwest to western Mongolia, and from the Qinghai-Tibet Plateau to the Yunnan-Guizhou Plateau in China (Zhu 1989; Wu and Wu 1992; Prokofiev 2017).

Syr Darya is the longest river in Central Asia and the second largest in volume after Amu Darya. It originates in the Fergana Valley at the confluence of the Naryn and Kara Darya, which flow from the Tian Shan Mountains and drain into the Aral Sea after passing through Uzbekistan, Tajikistan, and Kazakhstan. To date, eight species of *Triplophysa* species have been reported from the Syr Darya basin (Berg 1949; Turdakov 1963; Mitrofanov 1989; Sheraliev and Peng 2021a, b). The Fergana Valley is a mountainous region and its fish fauna differs from that of other regions in Uzbekistan. *Triplophysadorsalis* (Kessler, 1872), *T.elegans* (Kessler, 1874), *T.ferganaensis* Sheraliev & Peng, 2021, and *T.strauchii* (Kessler, 1874) have been recorded from the rivers of the Fergana Valley (Turdakov 1963; Sheraliev and Peng 2021b). The occurrence of *T.stolickai* (Steindachner, 1866) in the Fergana Valley is controversial and requires additional in-depth taxonomic research (Sheraliev and Peng 2021b; Sheraliev and Kayumova 2022).

The Sokh River is a tributary of the Syr Darya. It flows through the Sokh exclave of Uzbekistan, which is surrounded by Kyrgyzstan, and enters the Fergana Region. At present, the river fails to reach Syr Darya because its water is used for irrigation. The ichthyofauna of the Sokh River is almost unexplored. Here we report a new species of loach from the Sokh River.

## ﻿Materials and methods

### ﻿Specimen sampling, preservation, and morphological analysis

Handling of specimens was consistent with the Republic of Uzbekistan Animal Welfare Laws (No. 545-I 26.12.1997; https://lex.uz/docs/-31719), guidelines, and policies approved by the Southwest University Local Ethics Committee for Animal Experiments. After euthanasia, specimens were fixed in 10% formalin and stored in 70% ethanol. The right-side pectoral fin was preserved in 95% ethanol for molecular analysis. Counts and measurements were performed following the procedures of Kottelat and Freyhof (2007) and, whenever possible, on the left side of the specimen. Measurements to the nearest 0.01 mm were acquired using digital calipers and performed point-to-point rather than based on projections. The nomenclature of the head pores follows Kottelat (1984). Standard length was measured from the tip of the upper jaw to the end of the hypural complex, whereas the length of the caudal peduncle was measured from the base of the last ray of the anal fin to the end of the hypural complex at the mid-height of the base of the caudal fin. The last two branched rays in the dorsal and anal fins articulated on a single pterygiophore and were counted as single rays. Caudal-fin rays were counted separately in the upper and lower lobes; segmented unbranched and rudimentary rays were not counted. Cephalic lateral-line pores and gill rakers (four specimens) were counted under a stereo microscope (Nikon SMZ25, Tokyo, Japan). Vertebral counts (two specimens), including the four Weberian vertebrae, were obtained from x-radiographs. The specimens examined in the present study were deposited in the collections of the School of Life Sciences, Southwest University (SWU) in Beibei, Chongqing, China, and the private Bakhtiyor Sheraliev Fish Collection (BSFC) in Fergana, Uzbekistan.

Data on *Triplophysaparadoxa* (Turdakov, 1955), *T.ulacholica* (Anikin, 1905), and *T.coniptera* (Turdakov, 1954) were obtained from Turdakov (1963), *T.sewerzowi* (Nikolskii, 1938) from Mitrofanov (1989), *T.dorsonotata* (Kessler, 1879) and *T.lacusnigri* (Berg, 1928) from Prokofiev (2007), and *T.kungessana* (Kessler, 1879) from Zhao (1984). For *Triplophysa* species from the Tarim basin, data on *T.laterimaculata* Li, Liu & Yang, 2007, *T.papillosolabiata* (Kessler, 1879), *T.bombifrons* (Herzenstein, 1888), *T.zamegacephala* (Zhao, 1985), *T.moquensis* Ding, 1994, *T.orientalis* (Herzenstein, 1888), *T.microphysa* (Fang, 1935), and *T.incipiens* (Herzenstein, 1888) were obtained from Li et al. (2007). Data on *T.herzensteini* (Berg, 1909), *T.microphthalma* (Kessler, 1879), *T.kaznakowi* Prokofiev, 2004, and *T.waisihani* Cao & Zhang, 2008 were obtained from Cao and Zhang (2008). Other species used for comparative purposes were examined at BSFC, FSU, ICIZ, NWIPB, and SWU.

Abbreviations: **CPD**, caudal-peduncle depth; **CPL**, caudal-peduncle length; **HL**, head length; **K2P**, Kimura 2-parameter; **SL**, standard length.

Collection codes: **BSFC**, Bakhtiyor Sheraliev Fish Collection, Fergana, Uzbekistan; **FSU**, Fergana State University, Faculty of Life Sciences, Fergana, Uzbekistan; **ICIZ**, Ichthyological Collection of the Institute of Zoology, Academy of Sciences of Uzbekistan, Tashkent, Uzbekistan; **NWIPB**, Northwest Institute of Plateau Biology, Chinese Academy of Sciences, Qinghai, China; **SWU**, Southwest University, School of Life Sciences, Chongqing, China.

### ﻿DNA extraction, PCR amplification, and sequencing

DNA was extracted from the right-side pectoral fin using proteinase K digestion followed by a standard phenol-chloroform method (Sambrook and Russell 2001). The mitochondrial cytochrome *c* oxidase subunit 1 (COI) barcode region (652 bp) was amplified using primers VF2_t1 (5'-TGT AAA ACG GCC AGT CAA CCA ACC ACA AAG ACA TTG GCA C-3') and FR1d_t1 (5'-CAG GAA ACA GCT ATG ACA CCT CAG GGT GTC CGA ARA AYC ARA A-3'), as designed by Ivanova et al. (2007). The PCR assay was performed in a reaction volume of 25 µL containing 10 ng template DNA, 1 µL each primer, 12.5 µL 2× Taq Master Mix (Novoprotein, Guangdong, China), and double distilled water. Thermal cycling consisted of an initial step at 94 °C for 3 min, followed by 35 cycles at 94 °C for 20 s, 54 °C for 45 s, and 72 °C for 1 min 10 s, and a final extension step at 72 °C for 7 min. The PCR products were sent to TsingKe Biological Technology Co., Ltd (Chongqing, China) for sequencing.

### ﻿Phylogenetic reconstruction

The 652 bp COI gene sequence was used for phylogenetic analysis. Molecular analysis was conducted using three new COI sequences (*T.daryoae*, OK377300; *T.elegans*, OK377301; and *T.uranoscopus* (Kessler, 1872), OK377302), as well as 29 previously published sequences retrieved from the National Center for Biotechnology Information (NCBI) GenBank (Table 1) database (https://www.ncbi.nlm.nih.gov). The COI sequences were aligned using the Clustal_W algorithm in MEGA7 (Kumar et al. 2016), with manual checks for inconsistencies. The distances between different groups were determined using MEGA7, with 1000 bootstrap replicates calculated using the best-selected K2P model. For phylogenetic reconstruction, the datasets were analyzed based on Bayesian inference (BI) using MrBayes ver.3.2 (Ronquist et al. 2012) and maximum likelihood (ML) using MEGA7. MrBayes was run with six substitution types (nst = 6), considering a general time-reversible model with gamma-distributed rate variation and proportion of invariable sites (GTR+G+I) for the COI datasets. For BI analysis, we ran four simultaneous Monte Carlo Markov chains for 3,000,000 generations, with sampling every 1000 generations. The chain temperature was set as 0.2. Log-likelihood stability was determined after 10,000 generations, and the first 1000 trees were excluded as burn-in. The remaining trees were used to compute a 50% majority-rule consensus tree. For ML analysis, we conducted heuristic searches (1000 runs) also using the GTR+G+I model (nst = 6). Phylogenetic trees were visualized and edited using FigTree ver.1.4.2 (Rambaut 2014). *Barbatulabarbatula* (Linnaeus, 1758), (MF172073), and *B.toni* (Dybowski, 1869) (KX039660) were used as outgroup.

**Table 1.** List of mitochondrial COI sequences retrieved from GenBank with information on drainage and country of origin.

Species	Drainage	Country	GenBank Accession No.	Voucher ID	Reference
*Barbatulabarbatula*	Helge	Sweden	MF172073	NRM:44850	Norén et al. (2017)
*Barbatulatoni*	Yenisey	Russia	KX039660	Tuva-38FB	Freyhof et al. (2016)
*Triplophysaalticeps*	Qihai Lake	China	KT213585	NWIPB1206002	Wang et al. (2016)
*Triplophysaanterodorsalis*	Yangtze	China	MF123324	IHB-Tran-001	Shen et al. (2019)
*Triplophysableekeri*	Anning	China	JQ686729	–	Li et al. (2016)
*Triplophysableekeri*	Daning	China	JX135578	–	Tang et al. (2013)
*Triplophysachondrostoma*	Tiangeli	China	KU557964	CH3	Li et al. (2017)
*Triplophysachondrostoma*	Tiangeli	China	KT213589	NWIPB1006052	Wang et al. (2016)
*Triplophysadaryoae* sp. nov.	Sokh	Uzbekistan	OK377300	SWU540	This study
*Triplophysadorsalis*	Irtysh	China	KT241024	–	Lei et al. (2016)
*Triplophysadorsonotata*	Kegen	Kazakhstan	KX039654	Kaz-2-1	Freyhof et al. (2016)
*Triplophysaelegans*	Chirchik	Uzbekistan	OK377301	SWU634	This study
*Triplophysaferganaensis*	Shakhimardan	Uzbekistan	MW854332	SWU209	Sheraliev and Peng (2021b)
*Triplophysaleptosoma*	Ganzi	China	KT213593	NWIPB1109002	Wang et al. (2016)
*Triplophysaleptosoma*	Heihe	China	KX213692	LZUTL12022	Zhang et al. (2017)
*Triplophysamarkehenensis*	Sichuan	China	KT213594	SCU1010706	Wang et al. (2016)
*Triplophysamoquensis*	Ruoergai	China	KT213597	SCU20130901	Wang et al. (2016)
*Triplophysaobscura*	Jialing	China	MT271397	GS0629	Wang et al. (2020)
*Triplophysaorientalis*	Tagong	China	KU558037	TG5	Li et al. (2017)
*Triplophysaorientalis*	–	China	KJ631323	–	Ma and Yang (unpublished)
*Triplophysascleroptera*	Baijia	China	KT213602	IHB201306600	Wang et al. (2016)
*Triplophysasellaefer*	Juma	China	KY851112	IHB20151303	Feng et al. (2019b)
*Triplophysasewerzowi*	Kegen	Kazakhstan	KX039659	Kaz-2-2	Freyhof et al. (2016)
*Triplophysastolickai*	Zequ	China	KU558119	SST-2	Li et al. (2017)
*Triplophysastolickai*	–	China	JQ663847	–	Li et al. (2013)
*Triplophysastolickai*	Yangtze	China	MF123391	IHB-Trst-024	Shen et al. (2019)
*Triplophysastrauchii*	Chirchik	Uzbekistan	MW854336	SWU625	Sheraliev and Peng (2021b)
*Triplophysatenuis*	Dang	China	KT224363	IHB201307126	Wang et al. (2016)
*Triplophysaulacholica*	Mulei	China	KT259194	IHB201305179	Wang et al. (2016)
*Triplophysauranoscopus*	Zeravshan	Uzbekistan	OK377302	SWU524	This study
*Triplophysawuweiensis*	Jinchuanxia	China	KT224365	IHB201307124	Wang et al. (2016)
*Triplophysaxichangensis*	Anning	China	KT224366	IHB201306572	Wang et al. (2016)

## ﻿Results

### ﻿ Triplophysa daryoae sp. nov.

Taxon classificationAnimaliaCypriniformesNemacheilidae

E79D7931-3079-5A87-9FFE-2BED7C63B180

http://zoobank.org/8CE5BCB5-F671-4270-BFA3-7884DEF0BED7

**Figure 1.**
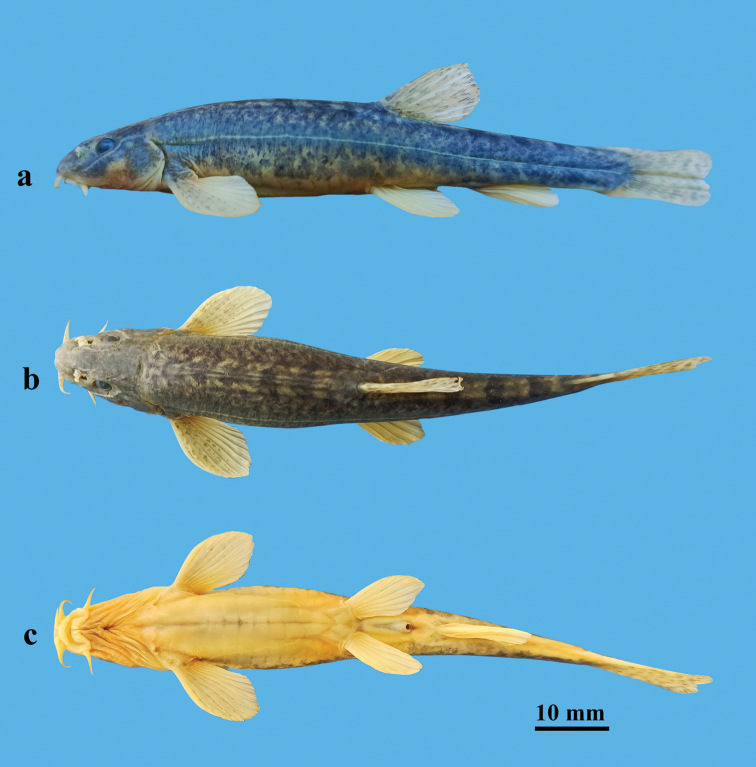
Lateral **a** dorsal **b** and ventral **c** views of *Triplophysadaryoae*, holotype, SWU 20211207001, male, 78.5 mm SL; Uzbekistan: Sokh River.

**Figure 2.**
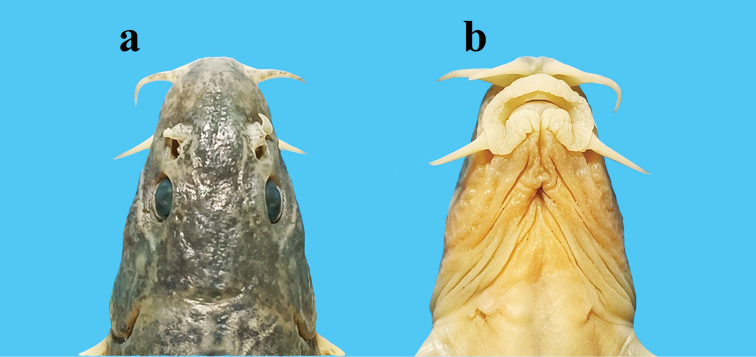
Dorsal **a** and ventral **b** views of the head of *Triplophysadaryoae*, SWU 20211207001, holotype, male, 78.5 mm SL.

**Figure 3.**
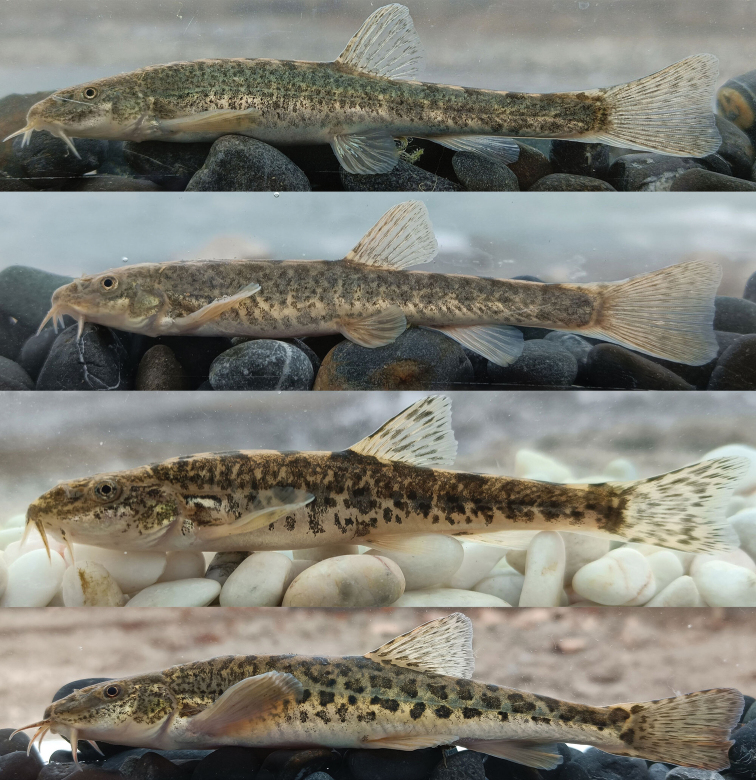
From top: *Triplophysadaryoae*, holotype SWU 20211207001, male, 78.5 mm SL, photographed alive immediately upon capture, Uzbekistan: Sokh River; *T.daryoae*, paratype, BSFC 0024, 72.8 mm SL, Uzbekistan: Sokh River; *T.ferganaensis*, BSFC 0025, 66.2 mm SL, Uzbekistan: Shahimar-dan stream; *T.strauchii*, not preserved, about 110 mm SL, Uzbekistan: Oltiariqsoy stream.

Figs 1 , 2 , 3 English common name: Sokh stone loach Uzbek common name: So‘x yalangbalig‘i Russian common name: Сохский голец

#### Holotype.

SWU 20211207001, male, 78.5 mm SL; Uzbekistan, Fergana Region, Sokh District, Sokh River, near Limbur village, an exclave of Uzbekistan surrounded by Kyrgyzstan, Syr Darya basin, 40°3.1528'N, 71°5.8195'E, altitude 1054 m, December 07, 2021, collected by B. Sheraliev and Y. Kayumova.

#### Paratypes.

SWU 20211207002-011, 10, 49.0–94.0 mm SL; BSFC 0023, 4, 62.1–82.4 mm SL; Uzbekistan, Fergana Region, Sokh District, Sokh River, near the Limbur village, exclave of Uzbekistan, Syr Darya basin, 40°2.7387'N, 71°6.288'E, altitude 1054 m, April 12, 2021, collected by Y. Kayumova. BSFC 0024, 3, 74.1–81.3 mm SL, same data as holotype.

#### Diagnosis.

*Triplophysadaryoae* is distinguished from congeners by a combination of characters. It is distinguished from *T.ferganaensis* by possessing a truncate caudal fin with 13–14 branched rays (vs emarginate, 16 rays), 9 pores in the pre-opercular mandibula (vs 7–8), and a slenderer body (body depth at dorsal-fin origin 1.4–1.8 times the HL vs 1.2–1.4). It is distinguished from *T.strauchii* by absence of the posterior chamber of the air bladder (vs developed, with a long tube), possessing 9–10 inner gill rakers on the first gill arch (vs 12–16), and no obvious skin mottling (vs mottling). *Triplophysadaryoae* is also distinguished from *T.dorsalis*, *T.dorsonotata*, and *T.elegans* by having a truncate caudal fin (vs emarginate) and lacking a posterior chamber of the air bladder (vs developed in *T.dorsalis* and *T.elegans*). It is distinguished from *T.sewerzowi*, *T.tenuis*, and *T.ulacholica* by the dorsal-fin origin opposite to the pelvic-fin insertion (vs anterior to vertical line of pelvic fin origin).

#### Description.

Morphometric data of *T.daryoae* are given in Table 2. Dorsal-fin rays iii, 6(2) or 7(16); anal-fin rays ii, 5; pectoral-fin rays i, 9(1), 10(16), or 11(1); pelvic-fin rays i, 6; caudal-fin rays, 13–14 (6+7 [5]; 7+7 [13]); vertebrae, 4+35 (*N* = 2); gill rakers, 9–10 in the inner row of first gill arch (*N* = 4). Cephalic lateral-line system, 2 supratemporal, 6 supraorbital, 4+10–11 infraorbital, and 9 pre-operculum mandibular pores.

**Table 2.** Morphometric data of *Triplophysadaryoae* (holotype SWU 20211207001, paratypes SWU 20211207002–011, *N* = 10; BSFC 0023, *N* = 4; BSFC 0024, *N* = 3) and closely related and occurred two loach species.

	*Triplophysadaryoae* sp. nov.				*Triplophysaferganaensis*				*Triplophysastrauchii*		
	holotype	holotype, paratypes (*N* = 18)			holotype	holotype, paratypes, non-types (*N* = 33)			range	(*N* = 9)	SD
		range	mean	SD		range	mean	SD		mean	
Standard length (mm)	78.54	49.00–94.04				42.85–109.17			83.63–155.86		
**In percent of standard length**											
Lateral head length	21.80	20.10–23.01	21.71	0.67	21.91	20.21–24.53	22.12	1.21	22.84–24.36	23.64	0.55
Body depth at dorsal-fin origin	15.33	12.37–15.33	13.91	0.76	16.03	14.57–17.39	15.85	0.72	19.23–19.91	19.63	0.27
Body width at dorsal-fin origin	11.85	10.84–12.72	11.81	0.54	13.40	11.99–15.93	13.37	0.87	16.72–18.43	17.44	0.62
Pre-dorsal length	55.03	51.32–55.03	53.52	1.04	52.34	49.35–56.80	53.36	1.56	51.51–53.62	52.51	0.82
Post-dorsal length	37.08	34.73–40.96	37.53	1.34	37.37	33.75–39.56	36.64	1.15	35.00–38.62	36.50	1.07
Pre-pelvic length	53.50	50.74–55.49	53.01	1.29	50.56	49.80–54.19	51.93	1.10	53.21–54.80	54.24	0.47
Preanal length	71.68	69.30–73.75	71.42	1.17	70.15	66.41–73.45	70.36	1.55	69.65–72.68	71.09	1.05
Preanus length	65.58	64.33–68.73	66.67	1.22	65.07	62.27–68.74	65.71	1.52	66.01–69.08	67.81	1.05
Dorsal-fin depth	16.51	14.85–18.54	16.22	0.87	16.14	13.61–19.08	16.88	1.48	17.07–19.80	18.10	0.83
Dorsal-fin base length	11.08	9.61–11.85	10.58	0.58	12.13	10.84–13.23	12.04	0.69	12.18–13.98	12.98	0.62
Anal-fin depth	16.68	13.09–16.68	14.66	0.86	15.14	12.67–18.27	15.17	1.35	13.38–15.81	14.33	0.89
Anal-fin base length	7.89	7.22–8.25	7.75	0.33	7.84	7.47–10.51	8.69	0.80	7.70–9.15	8.16	0.46
Pectoral-fin length	17.93	15.37–19.74	17.28	1.13	18.77	15.64–21.52	18.22	1.73	15.42–18.93	16.75	1.09
Pelvic-fin length	14.65	13.68–17.04	15.04	0.90	16.58	13.47–17.49	15.65	1.10	15.19–16.84	16.05	0.57
Caudal-fin length	21.86	18.98–23.60	20.91	1.43	21.99	19.62–25.25	22.07	1.51	19.30–22.80	20.69	1.27
Caudal-peduncle length (CPL)	23.07	19.12–23.07	20.80	1.11	20.50	18.45–23.11	20.72	1.19	18.70–23.39	20.85	1.35
Caudal-peduncle depth (CLD)	7.93	7.56–9.16	8.18	0.45	8.19	7.42–9.67	8.60	0.49	6.67–7.68	7.32	0.31
Pectoral-pelvic distance	32.77	30.37–34.18	32.09	1.11	30.92	28.80–35.29	31.62	1.45	31.09–33.84	32.48	0.84
Pelvic-anal distance	17.35	17.35–20.66	18.96	0.86	20.23	16.80–21.37	18.97	1.05	16.84–19.59	17.77	0.90
Vent – anal-fin origin distance	5.46	3.72–5.80	4.81	0.56	4.77	3.64–5.54	4.63	0.46	3.16–4.54	3.84	0.40
CPL/CPD	2.91	2.20–2.91	2.55	0.17	2.50	2.06–2.76	2.42	0.18	2.49–3.11	2.85	0.17
**In percent of head length**											
Head depth at nape	56.19	52.06–60.64	56.53	2.47	53.34	52.21–65.53	57.42	3.54	56.57–65.04	61.07	3.01
Head depth at eye	44.22	36.31–49.48	44.05	2.85	44.62	42.39–54.73	47.51	2.94	42.87–48.64	45.39	1.90
Maximum head width	70.74	63.20–73.16	67.78	3.51	68.68	59.87–79.24	68.84	4.23	63.36–70.59	67.70	2.32
Snout length	44.51	34.88–47.34	41.28	3.03	38.94	32.83–42.79	39.05	2.14	37.62–43.57	40.27	2.08
Eye diameter	13.26	12.49–17.08	14.07	1.51	13.73	10.33–17.03	13.86	1.37	12.49–16.28	14.04	1.30
Interorbital width	30.61	29.51–35.60	32.15	1.83	30.58	27.40–35.69	31.45	1.80	30.56–35.42	33.05	1.66
Postorbital distance	44.22	41.40–47.78	44.54	1.83	43.11	42.60–48.35	45.45	1.71	39.01–44.65	42.30	1.80
Maxillary barbel length	25.99	22.04–37.40	30.54	4.07	29.54	22.65–37.37	30.42	3.79	30.93–38.20	34.25	2.41
Inner rostral barbel length	22.55	19.82–30.17	24.18	2.68	25.37	19.31–27.62	23.63	2.33	23.06–30.14	26.63	2.27
Outer rostral barbel length	32.71	22.71–42.04	33.03	4.50	36.64	24.45–42.34	34.48	4.34	32.78–43.43	38.99	3.36

Body elongate; posterior portion gradually compressed from dorsal fin to caudal-fin origin. Dorsal profile slightly convex from the snout to the insertion of the anterior dorsal fin (Fig. 1). Deepest point of body slightly anterior to dorsal-fin origin; body depth at dorsal-fin origin 12.4–15.3% of SL. Head compression, maximum width always greater than depth; head maximum width 63.2–73.2% of HL. Snout slightly pointed, length shorter, equal, or slightly longer than postorbital length; snout length 34.9–47.3% of HL. Anterior and posterior nostrils adjacent; anterior nostril as short tube with elongated barbel-like tip; tip of nostril barbel not reaching the anterior margin of eyes. Eyes normal; diameter 12.5–17.1% of HL (Fig. 2). Mouth inferior, gape arched; mouth width 16.1–24.3% of HL. Rictus situated below the anterior nostril. Lips thick with furrows and papillae; upper lip pectinate, without medial notch; lower lip wide, interrupted in middle, with mental lobes and two highly developed ridges. Upper jaw covered by the upper lip; processus dentiformis absent. Three pairs of barbels: inner rostral barbel reaching rictus, length 19.8–30.2% of HL; outer rostral barbel reaching anterior margin of eye, length 22.7–42.0% of HL; maxillary barbel reaching posterior margin of eye, length 22.0–37.4% of HL.

Dorsal fin convex, origin opposite to pelvic-fin insertion, situated slightly posterior to midpoint between snout tip and caudal-fin base; upper margin slightly convex; second branched ray longest; depth of dorsal fin always shorter than lateral head length; depth 14.9–18.5% of SL. Anal fin short-based, posterior margin convex; length 13.1–16.7% of SL. Pectoral fins developed; 46.6–61.6% of pectoral-pelvic distance. Tips of depressed pelvic fins reaching the anus and anus separated from the anal-fin origin by a short distance. Caudal peduncle compressed laterally; length 2.2–2.9 times the peduncle depth. Caudal fin truncate, tips rounded; length 86.2–119.9% of caudal-peduncle length.

Body smooth and scaleless; cephalic lateral-line system well developed. Infraorbital and supraorbital canals stretching from the outer rostral barbel base and ethmoid, respectively, uniting in the posterior orbital region and extending posteriorly before converging with the supratemporal canal on the back of the head, and uniting with the lateral canal. Complete lateral line ending at caudal-fin base. Intestine moderately long, with two coils. Stomach U-shaped. Posterior chamber of the air bladder degenerated.

#### Coloration.

Dorsal profile grayish-brown to pale green without regular blotches in live individuals, and dark gray-brown in preserved specimens. Ventral side of the body ivory with gray tint. Dorsal side of head with small irregular dark melanophores; dorsal side of caudal peduncle with four or five irregular dark brown blotches. All fin membranes hyaline and light gray, without obvious mottling (Figs 1, 3).

#### Sexual dimorphism.

Mature males presenting granular tubercles on each side of the preorbital region and broadened and thickened external branched pectoral-fin rays dorsally covered by small and condensed epidermal breeding tubercles. Females without tubercles on the head and pectoral-fin rays.

#### Distribution and habitat.

**Figure 4.**
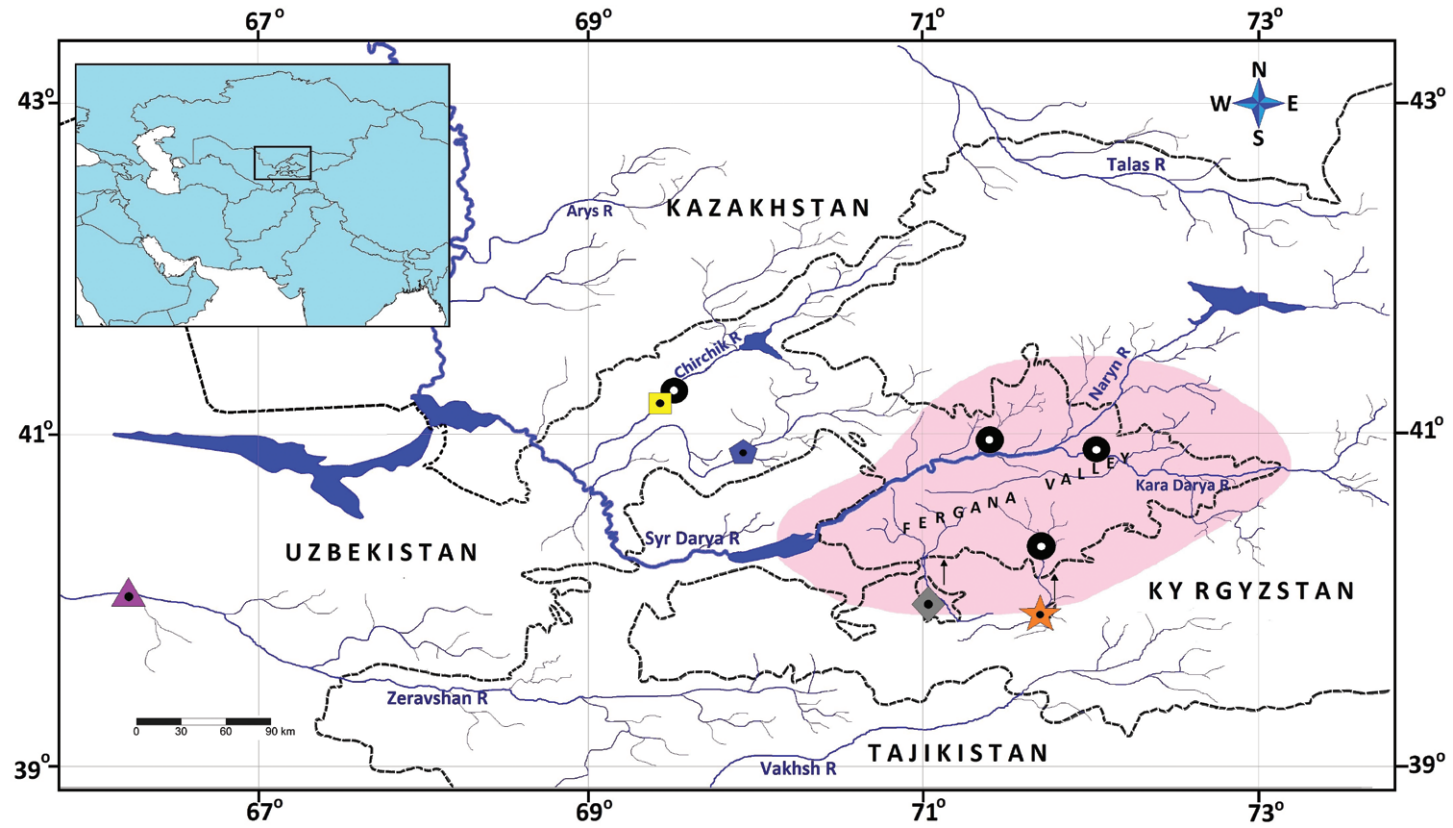
Map of the distribution of *Triplophysa* species in Uzbekistan: *T.daryoae* (grey diamond); *T.ferganaensis* (orange star); *T.strauchii* (black circle); *T.dorsalis* (blue pentagon); *T.elegans* (yellow rectangle); and *T.uranoscopus* (purple triangle).

**Figure 5.**
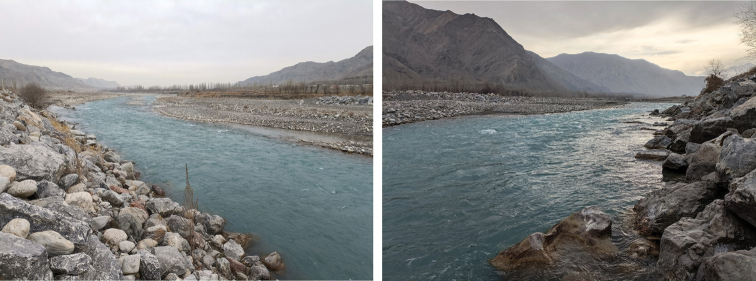
Sampling locality of the holotype (SWU 20211207001) of *Triplophysadaryoae* in the Sokh River left tributary of the Syr Darya, in Sokh District, the exclave of Uzbekistan, surrounded by Kyrgyzstan, photograph taken on December 7, 2021.

*Triplophysadaryoae* sp. nov. is known only from its type locality, the Sokh River, which originates in the Alay mountains and Turkestan range (Fig. 4). Presently, Sokh River water is primarily used for irrigation and does not reach Syr Darya. The river is located at an altitude of 700–1500 m and is constantly flowing rapidly; the water is clear and cold (the water temperature was 7.3 °C when the holotype was caught), and the bottom consists of gravel and stone (Fig. 5). *Triplophysadaryoae* cohabited with *Cottusspinulosus* Kessler, 1872 and *Schizothoraxeurystomus* Kessler, 1872, which are high-altitude fish species.

#### Etymology.

*Triplophysadaryoae* is dedicated to Daryo Sheralieva, the lovely daughter of the first author. The specific name is a noun in the genitive case.

##### ﻿Molecular analysis

**Figure 6.**
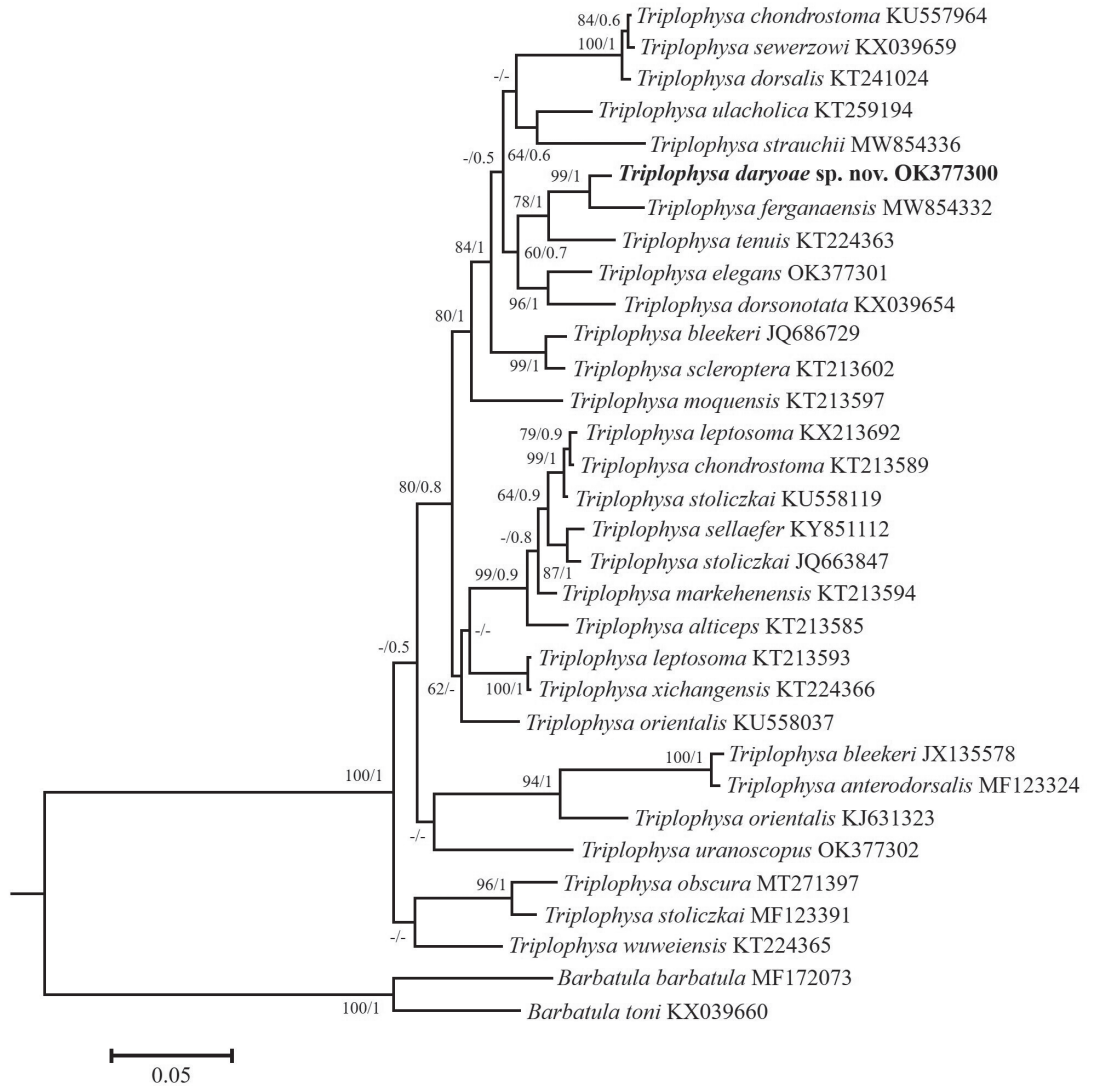
Bayesian inference tree based on mitochondrial COI gene sequences of 24 *Triplophysa* species. Maximum likelihood and Bayesian inference analyses resulted in congruent trees. Bootstrap and posterior probability values are shown above nodes on tree if 50% or higher.

COI sequence data (Fig. 6) showed that *Triplopysadaryoae* belongs to a group of species with a wide distribution in the Syr Darya, Tarim, and Ili-Balkhash river drainages, an endorheic basin in Central Asia. This group is defined here as the *T.dorsalis* species group, and our molecular data suggest that it includes *T.chondrostoma* (Herzenstein, 1888), *T.dorsalis*, *T.dorsonotata*, *T.elegans*, *T.ferganaensis*, *T.sewerzowi*, *T.strauchii*, *T.tenuis* (Day, 1877), and *T.ulacholica*. The minimum K2P distances between *T.daryoae* and its closest relatives *T.ferganaensis* and *T.tenuis* were 2.8% and 4.5%, respectively (Table 3). *Triplophysadaryoae* was distinguished from its most closely related congener, *T.ferganaensis*, by 18 unique and diagnostic nucleotide substitution sites in the COI barcode region (652 bp) (Table 4).

**Table 3.** The Kimura’s 2-parameter distance of mitochondrial COI dataset within *Triplophysadorsalis* species group based on 1000 bootstrap replications.

		1	2	3	4	5	6	7	8	9
**1**	*T.chondrostoma*									
**2**	*T.daryoae* sp. nov.	0.065								
**3**	*T.dorsalis*	0.003	0.065							
**4**	*T.dorsonotata*	0.081	0.062	0.077						
**5**	*T.elegans*	0.065	0.056	0.062	0.041					
**6**	*T.ferganaensis*	0.079	0.028	0.079	0.070	0.061				
**7**	*T.sewerzowi*	0.002	0.067	0.005	0.083	0.067	0.081			
**8**	*T.strauchii*	0.074	0.068	0.074	0.081	0.074	0.084	0.075		
**9**	*T.tenuis*	0.073	0.045	0.077	0.065	0.060	0.056	0.075	0.077	
**10**	*T.ulacholica*	0.053	0.063	0.056	0.062	0.060	0.075	0.055	0.058	0.061

**Table 4.** Diagnostic nucleotide substitutions in the 652 base pairs long mitochondrial COI barcoding region of *Triplophysadaryoae* and its closest two species.

**Species**	Variable Nucleotide Positions*																			
	90	117	120	123	129	153	210	249	255	264	267	270	273	279	288	291	306	315	318	334
*T.daryoae*	**G**	T	G	**A**	G	**C**	G	T	**A**	A	**T**	A	T	A	T	G	C	**A**	**C**	**C**
*T.ferganaensis*	**A**	T	G	**G**	G	**T**	G	T	**G**	A	**C**	A	T	A	T	G	C	**G**	**T**	**T**
*T.tenuis*	G	C	A	G	A	T	A	C	A	G	C	G	C	C	A	A	T	A	G	C
**Species**	**Variable Nucleotide Positions**																			
	375	411	453	462	465	468	471	510	547	558	561	570	582	585	589	603	606	666	678	699
*T.daryoae*	**A**	**C**	C	T	A	**A**	C	C	T	G	**T**	**G**	**A**	A	**T**	C	G	**T**	**G**	**C**
*T.ferganaensis*	**G**	**T**	C	T	A	**G**	C	C	T	G	**C**	**A**	**G**	A	**C**	C	G	**A**	**A**	**T**
*T.tenuis*	A	C	T	C	G	A	T	T	C	A	T	G	A	C	C	T	A	T	G	T

* The nucleotide position number was provided relative to the first nucleotide base of the complete COI gene of *T.tenuis* (KT224363).

## ﻿Discussion

*Triplophysa* differs from other Nemacheilidae genera by presenting sexual dimorphism (Zhu 1989). The presence of specific aggregations of breeding tubercles on the dorsal surfaces of the pectoral fin and from the lower edge of the eye to the base of the outer barbel in mature males can be regarded as an autapomorphy and is the unique diagnostic character of *Triplophysa* (Prokofiev 2010). These tubercles are present in *T.daryoae* males.

This study added three species (*T.daryoae*, *T.elegans*, and *T.uranoscopus*) to the previously published molecular reconstructions (Wang et al. 2016; Feng et al. 2019a; Wu et al. 2020). Our phylogenetic analysis was consistent with the results of previous molecular and morphological studies. Overemphasis on some characteristics with eco-phenotypic variation, such as body color, color patterns, barbel length, and mouth structure, when identifying *Triplophysa* species may be misleading (Ren et al. 2018). Therefore, it is advisable to employ a combination of morphological and molecular approaches to distinguish between loach species (Chen et al. 2021; Sheraliev and Peng 2021b; Deng et al. 2022; Lu et al. 2022).

*Triplophysadorsalis* is recorded from the middle and upper reaches of Kara Darya, whereas *T.elegans* is only recorded from the upper reaches (Baltabaev 1971). *Triplophysastrauchii* occurs in all waters of the Fergana Valley, whereas *T.ferganaensis* has only been recorded from its type locality, the Shakhimardan stream (Sheraliev and Peng 2021b). These species are more similar to *Triplophysadaryoae* than to other congeners. Nevertheless, they can be easily distinguished from the new species based on morphology.

*Triplophysadaryoae* can be distinguished from *T.ferganaensis*, which is the most similar species in terms of morphometric characteristics and habitat conditions, using the following characteristics: caudal fin truncate with 13 or 14 branched rays (vs emarginated with 16 branched rays), 6 branched pelvic-fin rays (vs 7 or 8), 9–11 (modally 10) branched pectoral-fin rays (vs 11–13, modally 12); cephalic lateral-line system with 6 suborbital and 9 pre-operculo mandibular pores (vs 7 and 7 or 8, respectively), dorsal and caudal fins almost hyaline, and spots imperceptible (vs spots on dorsal and caudal fins clearly visible). *Triplophysadaryoae* is distinguished from *T.strauchii*, which is the most common species of *Triplophysa* in the waters of Fergana Valley, by the small number of vertebrae (4+35 vs 4+37–38); a smaller number of gill rakers in the inner row of the first gill arch (9 or 10 vs 12–16); absent posterior chamber of air bladder (vs developed, with a long tube); shorter body depth and width at dorsal-fin origin (12.4–15.3% and 10.8–12.7% of SL vs 19.2–19.9% and 16.7–18.4% of SL, respectively); dorsal-fin origin equal to pelvic-fin insertion (vs anterior to vertical line of pelvic fin); and upper and lower lobes of caudal fin equal (vs upper lobe slightly longer than lower lobe). *Triplophysadaryoae* differs from *T.dorsalis* and *T.elegans*, which are rare species in the Fergana Valley, according to the following characteristics: dorsal-fin origin equal to pelvic-fin insertion (vs anterior to vertical line of pelvic fin in both); 9 or 10 gill rakers in the inner row of the first gill arch (vs 13–16 in *T.dorsalis*); wider interorbital width (29.5–35.6% of HL vs 23.9–27.6% of HL in *T.dorsalis*); longer pectoral-pelvic distance (30.4–34.2% of SL vs 24.6–28.7% of SL in *T.dorsalis*); shorter dorsal-fin depth (14.9–18.5% of SL vs 19.8–22.8% of SL in *T.dorsalis* and 18.9–24.1% of SL in *T.elegans*); caudal peduncle equal to HL (vs usually shorter in *T.dorsalis*); caudal-peduncle depth 7.6–9.2% of SL (vs 6.6–7.1% of SL in *T.elegans*); posterior chamber of air bladder degenerated (vs developed in both); lips thick with furrows (vs smooth lips in *T.dorsalis*); and caudal-peduncle depth 2.2–2.9 times its length (vs 3.2–3.5 times in *T.elegans*). Moreover, the genetic distance between the *TriplophysadaryoaeT.dorsalis*, *T.elegans*, *T.ferganaensis*, and *T.strauchii* (6.5%, 5.6%, 2.8%, and 6.8%, respectively), based on the mitochondrial COI barcoding region, is consistent with the species-level divergence in other fish taxa (Ward et al. 2005; Rosso et al. 2012; Abdulmalik-Labe and Quilang 2019; Freyhof et al. 2021).

Another ten species of *Triplophysa* occur in the Syr Darya basin and adjacent area of the Fergana Valley (Berg 1949; Turdakov 1963; Sheraliev and Peng 2021b). Among them, *T.tenuis* is similar to *T.daryoae* in its general body structure, especially in the caudal-fin shape. However, *T.daryoae* is distinguished from *T.tenuis* by presenting 9–10 gill rakers in the inner row of the first gill arch (vs 12–15); dorsal fin slightly posterior to the midpoint between the tip of the snout and caudal-fin origin (vs anterior to the midpoint between the tip of the snout and caudal-fin origin); caudal-peduncle depth 7.6–9.2% of SL (vs 5.2–5.7% of SL), head depth 52.1–60.6% of HL (vs 47.8–51.2% of HL); and caudal-peduncle depth 2.2–2.9 times its length (vs 4.1–4.3 times). *Triplophysadaryoae* is distinguished from *T.paradoxa* by the absence of scales (vs covered by scales), dorsal-fin origin opposite to pelvic-fin insertion (vs anterior to vertical line trough pelvic-fin origin), and dorsal-fin origin closer to the caudal-fin base than to the snout tip (vs closer to the snout tip). *Triplophysadaryoae* is distinguished from *T.ulacholica* by the shorter average caudal-peduncle length (20.8 ± 1.11% of SL vs 27.0 ± 0.24% of SL), deeper caudal peduncle (7.6–9.2% of SL vs 4.9–6.8% of SL), caudal-peduncle depth 2.2–2.9 times its length (vs 4.0–5.3 times), and smaller eye diameter (12.5–17.1% of HL vs 18.5–23.3% of HL). The new species can be distinguished from *T.coniptera* by the rounded edge of the pectoral fin (vs pointed); caudal fin truncate (vs deeply forked); snout length 7.4–10.0% of SL (vs 10.1–11.8% of SL); caudal-peduncle depth 7.6–9.2% of SL (vs 5.2–7.3% of SL); and caudal-peduncle depth 2.2–2.9 times its length (vs 3.4–4.5 times). *Triplophysadaryoae* is distinguished from *T.sewerzowi* by the following characteristics: dorsal-fin origin opposite to pelvic-fin insertion (vs anterior to vertical line of pelvic-fin origin), second branched ray of dorsal fin the longest (vs third or fourth), and degenerated posterior chamber of the air bladder (vs developed). It is also distinguished from *T.dorsonotata* by its smaller eye diameter (7.2 times of HL vs approximately 6 times), pre-dorsal length longer than post-dorsal length (vs pre-dorsal length slightly shorter than post-dorsal length), cephalic lateral-line system with 6 suborbital pores (vs 7 or 8), and caudal fin truncate (vs emarginated). The new species can be distinguished from *T.kungessana* by its thick lips with furrows and papillae (vs almost thin and smooth, without clear fringes or papillae) and pelvic fin reaching the anus (vs not reaching the anus). *Triplophysadaryoae* can be distinguished from *T.herzensteini* by having 4+35 vertebrae (vs 4+39–41) and a caudal-peduncle depth 7.6–9.2% of SL (vs 5.9–6.8% of SL). *Triplophysadaryoae* can be distinguished from *T.uranoscopus* by its caudal-peduncle depth measuring 2.2–2.9 times its length (vs. 2.7–4.1 times); caudal-peduncle depth 7.6–9.2% of SL (vs 5.4–7.0% of SL); dorsal-fin origin opposite to pelvic-fin insertion (vs anterior to vertical line of pelvic-fin origin). It differs from *T.lacusnigri* by a shorter head length (4.3–5.0 times SL vs 3.4–4.5 times SL) and non-oblique head profile in front of the eye (vs strongly oblique); the tip of the pectoral fin is usually formed by 4 branched rays (vs 2 or 3).

*Triplophysadorsonotata*, *T.elegans*, *T.lacusnigri*, *T.tenuis*, and *T.uranoscopus* from different water bodies in Central Asia have long been synonymized with *T.stolickai* due to their morphological resemblance (Berg 1949; Turdakov 1963; Zhu 1989; Wu and Wu 1992, Prokofiev 2010). We were unable to find *T.stolickai* in inland waters of Uzbekistan (Sheraliev and Peng 2021a). To confirm the existence of *T.stolickai* in the waters of Uzbekistan, further morphological and genetic taxonomic revisions of *Triplophysa* from this region, previously synonymized with *T.stolickai*, are required. However, Feng et al. (2019a) reported that *T.stolickai* from the Qinghai-Tibet Plateau represents an interesting case of morphological convergence and consists of distinct four lineages that are not closely related. In the phylogenetic tree presented here, *T.stolickai* nested in three lineages with genetic distances (K2P) from *T.daryoae* of 6.3%, 6.8%, and 9.7% (Fig. 6). Despite the high morphological diversity of *T.stolickai*, the new species differs from it by its caudal-peduncle depth (7.6–9.2% of SL vs 5.9–7.1% of SL in Ili River, 5.4–7.3% of SL in Tarim and Indus rivers, and 6.2–6.5% of SL in Yangtze River); interorbital width being 29.5–35.6% of HL (vs 20.8–26.2% of HL); head depth at nape 52.1–60.6% of HL (vs 46.1–51.5% of HL in Ili River); caudal-peduncle depth 2.2–2.9 times its length (vs 3.2–3.9 times in Ili River; 2.9–4.6 times in Tarim and Indus rivers; 3.6–3.8 times in Yangtze River); vertebrae 4+35 (vs 4+38–41); body without obvious mottling (vs. with mottling); supraorbital sensory canal always connected with the infraorbital canal (vs usually not connected); and 2 supratemporal pores (vs 3 or 4).

Berg (1905:243) noted that the rivers of the Syr Darya and Tarim basins originate on a flat marshy plateau in the Tian Shan, where water streams periodically change their direction and is difficult to distinguish between waters in separate basins. On this basis, he explained the similarity of ichthyofauna in the highlands of Central Asia (Berg, 1905). *Triplophysadaryoae* is similar to some species of *Triplophysa* from the Tarim basin and adjacent regions, with normal eyes, scaleless body, body color, color patterns, and slightly laterally compressed caudal peduncle (Li et al. 2007; Cao and Zhang 2008). However, it can be distinguished from *T.bombifrons*, *T.laterimaculata*, *T.moquensis*, *T.papillosolabiata*, and *T.zamegacephala* by the posterior chamber of its air bladder being degenerated (vs developed); 9 or 10 gill rakers in the inner row of the first gill arch (vs 12–14, 12, 13–15, 12–15, and 15–19, respectively); dorsal fin inserted opposite to vertical through pelvic-fin origin (vs dorsal-fin origin anterior to pelvic-fin insertion); pelvic fin reaching the anus (vs not reaching in *T.kaznakowi*); pre-pelvic length 50.7–55.5% of SL (vs 56.3–60.5% of SL in *T.laterimaculata*); caudal-peduncle length 19.1–23.1% of SL (vs. 28.6–31.3% of SL in *T.bombifrons* and 23.3–29.4% of SL in *T.papillosolabiata*); caudal-peduncle depth 7.6–9.2% of SL (vs. 4.5–6.7% of SL in *T.waisihani*); and 4+35 vertebrae (vs 4+38–43 in *T.moquensis* and 4+39–41 in *T.waisihani*). *T.daryoae* can be distinguished from other *Triplophysa* species by the following characteristics: 39 vertebrae (vs 41–47 in *T.orientalis*); caudal-peduncle depth 2.2–2.9 times its length (vs 3.3 times in *T.microphysa*, and 6.7–8.7 times in *T.incipiens*); and posterior chamber of air bladder degenerate (vs developed in *T.microphthalma*).

A *Triplophysadorsalis* species group is proposed here based on the molecular data and phylogenetic reconstruction obtained. The proposed species (see above) have also been nested in a single clade in previous phylogenetic studies of *Triplophysa* (Wang et al. 2016; Li et al. 2017; Feng et al. 2019a; Wu et al. 2020). However, no unique morphological synapomorphies that diagnose the *T.dorsalis* species group have been identified. In-depth morphological studies may clarify this issue in the future. Regarding biogeographical distribution, we hypothesize that species such as *T.coniptera* (Talas River), *T.dorsonotata* (Ili River), *T.herzensteini* (Ili River basin), *T.kungessana* (Künes River), *T.paradoxa* (Talas River basin) and *T.salari* (Chirchik River) also belong to this species group. In contrast, *T.uranoscopus*, which is widely distributed in the Zeravshan River, was not nested in this clade (Fig. 6). This result suggests that loaches from Amu Darya have evolved separately from the *Triplophysa* of Syr Darya. A comprehensive study of this situation by examination of other species of *Triplophysa* (e.g., *T.lacusnigri* and *T.kafirnigani*) in the Amu Darya basin may serve as an important key to understanding how *Triplophysa* species have evolved in Central Asia.

### ﻿Key to *Triplophysa* species occurring in the Syr Darya basin and adjacent regions

**Table:** 

1	The posterior chamber of the air bladder developed, clearly visible	**2**
–	The posterior chamber of the air bladder degenerated, directly connecting with the bony capsule	**4**
2	The caudal peduncle compressed at the base, its width less than its depth	***T.dorsalis* (Syr darya basin)**
–	The caudal peduncle not compressed at the base, its width greater than or equal to its depth	**3**
3	Branched dorsal-fin rays usually 8, caudal-fin emarginated and upper lobe longer than lower, maximum body depth fits to the origin of dorsal-fin	***T.strauchii* (Balkhash and upper Syr Darya basin)**
–	Branched dorsal-fin rays usually 7, caudal-fin truncate, upper and lower lobes equal, maximum body depth significantly anterior to the origin of dorsal-fin	***T.ulacholica* (Issyk Kul Lake and its tributaries)**
4	Caudal-fin truncate	**5**
–	Caudal-fin emarginated or forked	**7**
5	Standard length not exceeding 100 mm; brush-like agglomerations on the sides of the head are absent	***T.sewerzowi* (Ili River basin)**
–	Standard length exceeding 100 mm; brush-like agglomerations on the sides of the head are present	**6**
6	Gill rakers in the inner row on the first gill arch 9–10; caudal peduncle depth 3 times less than its length	***T.daryoae* sp. nov. (Sokh River)**
–	Gill rakers in the inner row on the first gill arch 12–15; caudal peduncle depth 4 times more than its length	***T.tenuis* (Yarkand River)**
7	Vertebrae number exceeds 40	**8**
–	Vertebrae number does not exceed 40	**11**
8	Branched pectoral-fin rays 10 or more; intestine not too long, with less than 6 loops	**9**
–	Branched pectoral-fin rays 10 or less; intestine too long, with 10 loops	***T.chondrostoma* (Qaidam basin)**
9	Caudal-fin forked; caudal peduncle depth more than 6% of SL	**10**
–	Caudal-fin emarginated; caudal peduncle depth less than 6% of SL	***T.dorsonotata* (Ili River basin)**
10	Branched dorsal-fin rays usually 8; cephalic lateral-line system with more than 14 infraorbital pores	***T.stenura* (Yangtze River basin)**
–	Branched dorsal-fin rays usually 7; cephalic lateral-line system with less than 14 infraorbital pores	***T.coniptera* (Talas River basin)**
11	Caudal peduncle depth more than 8% of SL; 8–9 light-brown irregular blotches on dorsum and 10–12 dark-grey spots on side	***T.ferganaensis* (Shakhimardan River)**
–	Caudal peduncle depth less than 7% of SL; 6–15 transverse stripes on side and back and brindle colored	***T.elegans* (Chirchik River)**

### ﻿Comparative materials

*T.dorsalis*: FSU uncatalogued, 6, 56.2–83.5 mm SL; Kara Darya, Andijan Region, Uzbekistan; ICIZ 2200016, 11, 51.6–88.8 mm SL; Achangaran River, Tashkent Region, Uzbekistan.

*T.elegans*: SWU 20190818630–634, 5, 47.9–69.1 mm SL; Chirchik River, Tashkent Region, Uzbekistan.

*T.ferganaensis*: SWU 20190813001, holotype, 87.5 mm SL; Shakhimardan stream in Yordon village, Syr Darya basin, Fergana District, Fergana Region, Uzbekistan. SWU 20190813002–021, FSU 082019650–654, 25 paratypes, 49.5–109.2 mm SL; Shakhimardan stream in Yordon village, Syr Darya basin, Fergana District, Fergana Region, Uzbekistan. BSFC 0025, 2, 42.9–66.2 mm SL; Shakhimardan stream in Yordon village, Syr Darya basin, Fergana District, Fergana Region, Uzbekistan.

*T.stolickai*: NWIPB 1305044, 1305046–48, 1305052, 1305056, 1305060, 7, 56.0–102.5 mm SL; Kashi River, Nilka County, Ili River System, Xinjiang Province, China. NWIPB 1305111, 1305113–115, 4, 67.6–91.0 mm SL; Künes River, Xinyuan County, Ili River System, Xinjiang Province, China. NWIPB 1305131, 1305141–142, 3, 64.0–75.4 mm SL; Tekes River, Tekes County, Ili River System, Xinjiang Province, China. NWIPB 1307006–007, 1307014, 3, 58.3–79.5 mm SL; Zhaqu River, Chindu County, Yangtze River System, Qinghai Province, China. NWIPB 1407013–018, 6, 62.7–84.2 mm SL; Changchuan River, Rutog County, Indus River System, Tibet Autonomous Region, China. NWIPB 1007083, 1, 97.5 mm SL; Yarkand River, Yecheng County, Tarim River System, Xinjiang Province, China. NWIPB 1007084, 1, 73.9 mm SL; Qaraqash River, Pishan County, Tarim River System, Xinjiang Province, China.

*T.strauchii*: SWU 20190820642–644, 3, 74.0–110.5 mm SL; unnamed stream, Syr Darya River System, Fergana District, Fergana Region, Uzbekistan. SWU 20190809551, 1, 69.5 mm SL; Kara Darya, Andijan Region, Uzbekistan. SWU 20190818617–642, 26, 45.9–98.7 mm SL; Chirchik River, Tashkent Region, Uzbekistan. BSFC 0022, 8, 83.6–155.9 mm SL; Great Fergana Canal, Syr Darya River System, Uzbekistan District, Fergana Region, Uzbekistan.

*T.tenuis*: NWIPB1250170–174, 5, 83.2–111.2 mm SL; Heihe River, Zhangye city, Heihe River System, Gansu Province, China.

*T.uranoscopus*: SWU 20190802503–504, 2, 78.0–80.2 mm SL; Zeravshan River, Samarkand Region, Uzbekistan; BSFC 0041, 8, 51.5–96.1 mm SL; Karadarya River, Zeravshan River System, Oqdaryo District, Samarkand Region, Uzbekistan.

## Supplementary Material

XML Treatment for Triplophysa daryoae
